# Differing Patterns of Overweight and Obesity among Black Men and Women in Cape Town: The CRIBSA Study

**DOI:** 10.1371/journal.pone.0107471

**Published:** 2014-09-15

**Authors:** Nasheeta Peer, Carl Lombard, Krisela Steyn, Nomonde Gwebushe, Naomi Levitt

**Affiliations:** 1 Non-communicable Diseases Research Unit, Medical Research Council, Durban, South Africa; 2 Chronic Disease Initiative for Africa, Department of Medicine, University of Cape Town, Cape Town, South Africa; 3 Biostatistics Unit, Medical Research Council, Cape Town, South Africa; 4 Division of Endocrinology and Diabetes, Department of Medicine, University of Cape Town, Cape Town, South Africa; University of Warwick – Medical School, United Kingdom

## Abstract

**Objectives:**

To ascertain the prevalence and determinants of overweight/obesity in the 25–74-year-old urban black population of Cape Town and examine the changes between 1990 and 2008/09.

**Methods:**

In 2008/09, a representative cross-sectional sample, stratified for age and sex, was randomly selected from the same townships sampled in 1990. Data were collected by questionnaires, clinical measurements and biochemical analyses. Gender-specific linear regression models evaluated the associations with overweight/obesity.

**Results:**

There were 1099 participants, 392 men and 707 women (response rate 86%) in 2008/09. Mean body mass index (BMI) and waist circumference (WC) were 23.7 kg/m^2^ (95% confidence interval (CI): 23.1–24.2) and 84.2 cm (95% CI: 82.8–85.6) in men, and 33.0 kg/m^2^ (95% CI: 32.3–33.7) and 96.8 cm (95% CI: 95.5–98.1) in women. Prevalence of BMI ≥25 kg/m^2^ and raised WC were 28.9% (95% CI: 24.1–34.3) and 20.1% (95% CI: 15.9–24.9) in men, and 82.8% (95% CI: 79.3–85.9) and 86.0% (95% CI: 82.9–88.6) in women. Among 25–64-year-olds, BMI ≥25 kg/m^2^ decreased between 1990 (37.3%, 95% CI: 31.7–43.1) and 2008/09 (27.7%, 95% CI: 22.7–33.4) in men but increased from 72.7% (95% CI: 67.6–77.2) to 82.6% (95% CI: 78.8–85.8) in women. In the regression models for men and women, higher BMI was directly associated with increasing age, wealth, hypertension and diabetes but inversely related to daily smoking. Also significantly associated with rising BMI were raised low-density lipoprotein cholesterol and being employed compared to unemployed in men, and having >7 years of education in women.

**Conclusions:**

Overweight/obesity, particularly in urban black women, requires urgent action because of the associations with cardiovascular disease risk factors and their serious consequences.

## Introduction

There has been a rapid rise in the prevalence of overweight and obesity with the emergence of an ‘obesogenic’ environment [1]. Globalisation and urbanisation have contributed to environmental and societal changes with subsequent alterations in dietary and physical activity patterns. Consequently, overweight/obesity has reached epidemic proportions, with obesity overtaking tobacco as the largest preventable cause of disease burden in some regions [2].

In South Africa, the proportions of diabetes, cardiovascular disease (CVD) and selected cancers attributable to excess body weight were higher than the global estimates, particularly in women [3]. Body mass index (BMI) >21 kg/m^2^ was responsible for 87% of diabetes, 68% of hypertensive disease, 38% of ischaemic heart disease and 45% of ischaemic stroke. In 2000, 7.0% of mortality in South Africa was attributable to excess body weight, with the burden approximately double in women than men [3].

The high burden attributable to excess body weight in the country underscores the need, particularly in an ‘obesogenic’ environment, for regular monitoring of adiposity. The most recent national anthropometric surveillance data was obtained in the 2012 South African National Health and Nutrition Examination Survey (SANHANES-1) [4] but on a regional level these have not been examined in the urban black population of Cape Town in almost two decades. The Cardiovascular Risk in Black South Africans (CRIBSA) study thus aimed to ascertain the prevalence and determinants of overweight/obesity in black men and women in Cape Town in 2008/09 and to compare these findings with a similar study conducted in 1990.

## Methodology

### Study population and sampling procedure

In 2008/09, a cross-sectional study was conducted among a random sample of 25–74-year-old men and women in the same predominantly black residential areas in Cape Town where a study was conducted in 1990. This was to ensure comparability with the latter, the methodology of which has been described previously [5] as has the sampling procedure for the current study [6]. Participants with known HIV infection or on tuberculosis treatment were among those excluded. The study was conducted during office hours and this may have contributed to selection bias because of the inclusion of fewer employed compared to unemployed participants, particularly among men.

### Data collection

Data, obtained via fieldworker administered questionnaires, included the Global Physical Activity Questionnaire (GPAQ) [7] and the CAGE set of four questions for determining problematic alcohol use [8]. Also recorded were assets defining wealth, including ownership of consumer items (durable goods), access to electricity, and the source of drinking water and toilet facilities.

Height, weight, and waist and hip circumferences were measured using standardised techniques [9]. Weight was measured to the nearest 0.5 kg, using a calibrated scale, with each participant barefoot and in light clothing. Height was measured to the nearest 0.1 cm using a stadiometer. A flexible tape measure measured waist and hip circumferences to the nearest 0.1 cm. Waist circumference (WC) was measured approximately 2 cm or two finger spacings above the umbilicus with a flexible tape measure held parallel to the floor. Hip circumference was measured at the maximum posterior protuberance of the buttocks while the participant was standing upright with feet together.

The average of the second and third of three blood pressure (BP) measurements taken two-minute apart using an Omron BP monitor were used in the analyses. Blood samples, for lipid and glucose estimations, were drawn following an overnight fast of 10 hours, and a standard oral glucose tolerance test administered with blood samples taken 120 minutes later [10].

### Definitions

The anthropometric indices computed BMI as weight in kilograms divided by height in metres squared (kg/m^2^). BMI was categorised as underweight (<18.5), normal weight (18.5–24.9), overweight (25–29.9) and obese (≥30). Raised WC (men: >94 cm, women >80 cm) and raised waist-to-hip ratio (WHR) (men: >1.0, women: >0.85) were identified using the World Health Organization (WHO) criteria [11].

Additional definitions were as follows: smoking status: smoking ≥1 cigarette a day, problematic alcohol use: ≥2 CAGE questions answered affirmatively [8], physical inactivity: <150 minutes of moderate to vigorous activity per week, and hypertension: BP ≥140/90 mmHg or using antihypertensive agents. Diabetes was diagnosed according to the 1998 WHO definition [10]. Low-density lipoprotein cholesterol (LDL-C) was calculated using the Friedewald equation with raised levels identified as LDL-C >3.0 mmol/l [12].

### Statistical analyses

Data analyses were done using STATA 12. Descriptive statistics, including crude prevalence, were calculated using the weights based on the sample design and adjusted for the realised sample. A principal component analysis of the pooled data, based on the assets that defined wealth, was used to develop an asset index [13]. Categories of relative wealth were created using tertiles.

Survey-based odds ratio and 95% confidence interval (95% CI) for the associations of the socio-demographic variables and CVD risk factors with BMI ≥25 kg/m^2^ were calculated (unadjusted). These were entered in the linear regression models with BMI as the independent variable. Separate models for men and women were fitted due to significant interactions between socio-demographic variables and gender. To assess the associations of the socio-demographic variables and CVD risk factors with BMI and WC, survey linear regression analyses were performed; only the main effects were modelled. The data for BMI are presented and the differences in the associations for WC compared with BMI are described but have not been tabulated on account of the similarities.

The comparison between the 1990 and 2008/09 data was done for 25–64-year-olds (1990: n = 665; 2008/09: n = 1022) because these were the common age categories between the two studies. Also, the comparison is limited to BMI as waist and hip measurements were not taken in 1990. A direct comparison of the 2008/09 and 1990 datasets could not be conducted because of the geographic and demographic changes in some residential areas during this period with the populations increasing markedly and a concomitant expansion in area size. This necessitated the use of 95% CI for the comparison of the BMI data between the two surveys.

The University of Cape Town's Research and Ethics Committee approved the study. All participants signed informed consent.

## Results

The realised study sample comprised 1099 participants, 392 men and 707 women. Among 25–74-year-olds in 2008/09, mean BMI and WC levels were significantly higher (p<0.001) in women (33.1±8.1 kg/m^2^ and 97.5±15.0 cm) than in men (24.0±5.3 kg/m^2^ and 85.7±13.8 cm) (Data not shown). Similarly, the prevalence of obesity was higher in women (62.1%, 95% CI: 57.8–66.2) compared to men (10.3%, 95% CI: 7.4–14.2) (p<0.001) as was raised WC (women: 86.0%, 95% CI: 82.9–88.6; men: 20.1%, 95% CI: 15.9–24.9, p<0.001). Mean WHR was 0.89±0.1 in men and 0.85±0.1 in women (p<0.001) while raised WHR was lower in men (9.2%, 95% CI: 6.5–12.9) than in women (47.8%, 95% CI: 43.9–51.8) (p<0.001).

The comparison of BMI indices among 25–64-year-olds between 1990 and 2008/09 showed that mean BMI among men was similar, while among women it increased from 29.5 kg/m^2^ to 33.0 kg/m^2^ (**Table 1**). Between 1990 and 2008/09, overweight decreased in both men and women but obesity remained unchanged in men at 9.5% while increasing significantly in women from 42.7% to 61.5%.

**Table 1:** Body mass index (BMI) in 25–64-year-old men and women in 1990 and 2008/09.

	1990		2008/09	
		95%CI		95%CI
**Men (1990: n = 292; 2008/09: n = 364)**				
BMI (kg/m^2^), mean	24.3	23.8–24.8	23.6	23.0–24.1
BMI (kg/m^2^),%:				
Underweight (<18.5)	2.7	1.3–5.7	7.6	5.0–11.4
Normal weight (18.5–24.9)	60.0	54.1–65.7	64.8	58.9–70.2
Overweight (25–29.9)	27.8	22.8–33.4	18.2	14.3–23.0
Obese (≥30)	9.5	6.7–13.3	9.5	6.5–13.6
**Women (1990: n = 373; 2008/09: n = 658)**				
BMI (kg/m^2^), mean	29.5	28.8–30.2	33.0	32.3–33.7
BMI (kg/m^2^),%:				
Underweight (<18.5)	2.1	1.0–4.3	0.7	0.3–1.9
Normal weight (18.5–24.9)	25.3	20.9–30.2	16.8	13.7–20.3
Overweight (25–29.9)	30.0	25.3–35.1	21.1	17.7–25.0
Obese (≥30)	42.7	37.5–48.0	61.5	57.1–65.7
**Total (1990: n = 665; 2008/09: n = 1022)**				
BMI (kg/m^2^), mean	27.0	26.5–27.5	28.5	27.9–29.2
BMI (kg/m^2^),%:				
Underweight (<18.5)	2.4	1.4–4.0	3.9	2.7–5.8
Normal weight (18.5–24.9)	42.3	38.4–46.3	39.5	35.5–43.7
Overweight (25–29.9)	28.9	25.4–32.7	19.7	16.8–23.1
Obese (≥30)	26.4	23.2–29.9	36.8	33.0–40.7

**Figure 1:**
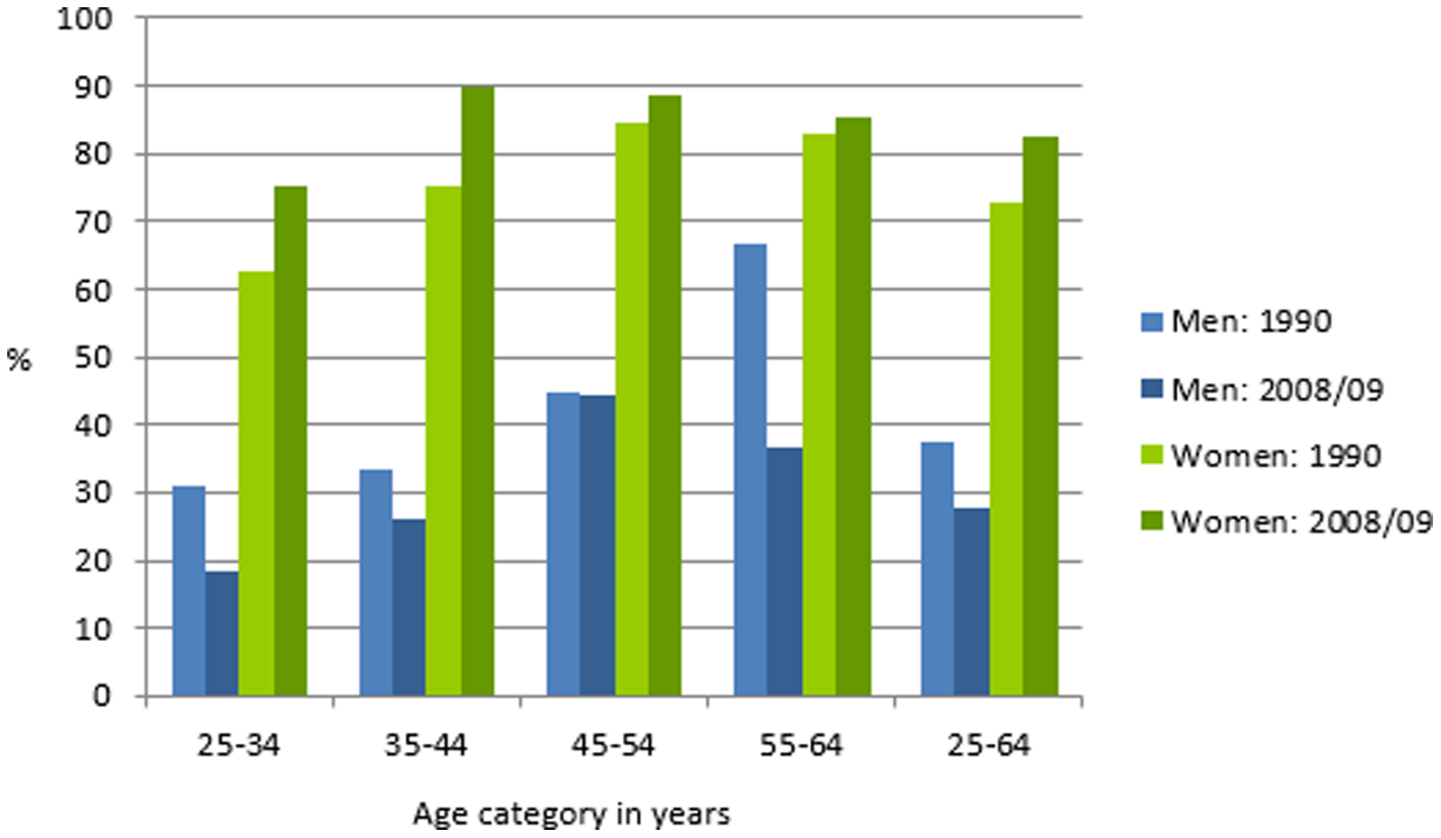
Prevalence of body mass index ≥25 kg/m^2^ in 25–64-year-old men and women in 1990 and 2008/09.

The most marked decline in overweight/obesity was in 55–64-year-old men from 66.7% in 1990 to 36.5% in 2008/09 **(** **Figure 1** **)**. Conversely, the most marked increases in overweight/obesity among women occurred in those younger than 45.

Overweight/obese men and women in 2008/09 were significantly older, more likely to be pensioners, lived in better quality housing and were wealthier than their counterparts (**Table 2**). Overweight/obese men, but not women, were also more likely to be employed than those with BMI <25 kg/m^2^. Hypertension, diabetes and raised LDL-C were more prevalent in men and women with, than without, overweight/obesity.

**Table 2:** Socio-demographic and cardiovascular disease risk factors in overweight/obese men and women in 2008/09.

	Overweight/obese Men				Overweight/obese Women			
	N	%	Odds Ratio	95%CI	N	%	Odds Ratio	95%CI
***Socio-demographic factors***								
Number	392	28.9			707	82.8		
Age in years: 25–34	121	18.5	1.00		236	75.3	1.00	
35–44	99	26.0	1.55	0.81–2.94	162	89.9	2.91	1.57–5.41
45–54	86	44.5	3.53	1.72–7.26	168	88.7	2.58	1.47–4.52
55–64	58	36.5	2.53	1.21–5.31	92	85.3	1.89	0.90–3.98
65–74	28	50.0	4.40	1.84–10.52	49	87.8	2.35	0.92–6.00
Education: ≤7 years	155	31.4	1.00		261	80.9	1.00	
>7 years	237	27.4	0.82	0.49–1.40	446	83.7	1.21	0.78–1.89
% of life spent in the city: <50	132	26.9	1.00		318	81.0	1.00	
≥50	260	27.0	1.16	0.70–1.94	389	84.4	1.27	0.80–2.00
Work: Unemployed	235	20.3	1.00		412	81.5	1.00	
Employed	91	41.5	2.79	1.60–4.87	141	82.3	1.05	0.60–1.85
Pensioners	40	54.6	4.73	2.04–10.97	112	89.9	2.01	1.03–3.93
Other*	26	27.9	1.52	0.60–3.83	42	85.0	1.29	0.50–3.34
House: Informal shack	190	24.2	1.00		289	78.9	1.00	
Council/core house/hostel	115	30.3	1.36	0.74–2.53	274	84.9	1.50	0.96–2.36
Built formal unit (private)	87	38.2	1.94	1.11–3.40	144	89.3	2.22	1.17–4.24
Wealth tertile: 1^st^ (lowest)	142	15.7	1.00		221	70.7	1.00	
2^nd^	115	26.6	1.94	1.07–3.51	264	85.2	2.38	1.45–3.91
3^rd^ (highest)	135	43.6	4.15	2.47–6.98	222	92.6	5.18	2.64–10.14
***CVD risk factors***								
Physical activity/week: ≥150minutes	365	28.3	1.00		657	83.1	1.00	
<150minutes	27	37.3	1.50	0.65–3.50	50	79.4	0.78	0.36–1.69
Dietary fat intake: <30%	260	27.9	1.00		444	80.4	1.00	
≥30%	132	30.8	1.15	0.73–1.80	263	87.1	1.65	1.02–2.65
Smoking ≥1 cigarette/day: No	209	42.7	1.00		649	84.5	1.00	
Yes	183	14.8	0.23	0.14–0.40	58	63.6	0.32	0.17–0.59
Alcohol use: CAGE <2	198	36.5	1.00		583	83.8	1.00	
CAGE ≥2	194	21.2	0.47	0.28–0.79	124	78.6	0.71	0.42–1.21
Hypertension: No	230	22.0	1.00		408	78.0	1.00	
Yes	162	40.4	2.41	1.47–3.93	299	90.9	2.84	1.67–4.83
Diabetes: No	347	25.9	1.00		592	80.3	1.00	
Yes	45	54.9	3.48	1.71–7.05	115	98.5	15.7	4.71–52.43
LDL-C >3 mmol/l: No	236	17.4	1.00		343	79.0	1.00	
Yes	156	47.8	4.35	2.68–7.06	364	87.2	1.81	1.18–2.79

*Other: comprised of homemakers, students and those receiving disability grants.

In the linear regression models for both men and women (**Table 3**), there were significant positive associations between BMI and age, relative wealth, hypertension and diabetes while there was an inverse relation with daily smoking. Additionally, in men, BMI was positively associated with being employed compared to unemployed and with raised LDL-C, and, in women, with >7 years of education.

**Table 3:** Gender-specific linear regression models of body mass index on socio-demographic and cardiovascular disease risk factors.

	Men (n = 392); R^2^ = 0.337				Women (n = 707); R^2^ = 0.140			
	Coefficient	95% Confidence Interval		p-value	Coefficient	95% Confidence Interval		p-value
		Lower limit	Upper limit			Lower limit	Upper limit	
Increasing age (years)	0.06	0.01	0.11	**0.010**	0.09	0.01	0.17	**0.027**
Education >7 years	−0.48	−1.63	0.67	0.410	1.92	0.58	3.26	**0.005**
≥50% of life spent in the city	−0.24	−0.98	0.50	0.528	−0.03	−1.34	1.27	0.960
Work:				**0.011**				0.814
Unemployed		1.00			1.00			
Employed	2.02	0.77	3.27	**0.002**	−0.58	−2.12	0.96	0.460
Pensioners	−0.34	−2.50	1.82	0.757	−0.92	−3.63	1.80	0.506
Other*	−0.16	−1.69	1.36	0.832	−0.83	−3.53	1.87	0.546
House:				0.864				0.271
Informal shack		1.00			1.00			
Council/core house/hostel	0.10	−0.97	1.17	0.857	−0.77	−2.31	0.77	0.327
Built formal unit (private)	−0.24	−1.45	0.97	0.693	−1.46	−3.25	0.32	0.107
Wealth tertile:				**<0.001**				**<0.001**
1^st^ (lowest)		1.00			1.00			
2^nd^	0.23	−0.82	1.27	0.668	3.09	1.61	4.57	**<0.001**
3^rd^ (highest)	2.30	0.96	3.64	0.001	4.93	3.19	6.66	**<0.001**
Dietary fat intake ≥30%	0.10	−0.79	0.99	0.828	0.86	−0.47	2.19	0.205
<150 min moderate-vigorous activity/week	1.29	−1.05	3.63	0.277	−0.48	−2.85	1.89	0.689
Smoking ≥1 cigarette/day	−2.14	−2.98	−1.31	**<0.001**	−3.14	−5.70	−0.58	**0.017**
Problem drinking (CAGE ≥2)	−0.34	−1.25	0.58	0.467	0.18	−1.66	2.01	0.849
Hypertension	0.92	0.06	1.78	**0.036**	2.67	0.89	4.46	**0.004**
Diabetes	2.20	0.43	3.97	**0.015**	1.79	0.21	3.37	**0.027**
LDL-C >3 mmol/l	1.92	1.07	2.76	**<0.001**	0.08	−1.19	1.35	0.900

*Other: housewife, student and those receiving disability grants.

When WC replaced BMI in the same models, there were no changes in the direction or significance of the associations in men. In women, however, living in informal dwellings compared to formal housing was significantly related to increasing WC (difference 36.30 cm, 95% CI 4.00–68.60 cm, p = 0.028); there was no change in the direction or significance of the other variables.

## Discussion

The prevalence of overweight/obesity, at 82.8% among black women in Cape Town in 2008/09, has reached epidemic proportions. Not only is this the highest reported in Sub-Saharan Africa (SSA) [14], [15], it is even markedly higher than in the United States (64.1% reported among ≥20-year-old women), a nation where overweight/obesity is known to be highly prevalent [16]. Moreover, the obesity rate in this study was 1.8 times greater than the 35.5% prevalence in American women [16] and considerably higher than the global average of 13.8% [17]. Notably, the mean BMI among women in this study was in the obese range (33.0 kg/m^2^) and approached that of women from Nauru who have the highest mean BMI (35.0 kg/m^2^) in the world [17]. Furthermore, abdominal obesity, as defined by central adiposity and measured by WC and WHR, was also the highest among women in this study compared to other SSA nations when using the same cut-off value [14], [18].

The socio-cultural determinants of obesity are complex and range from perceptions of what constitutes an ideal body shape and size, with the preferred form being overweight, to food availability and portion sizes [19]. Additionally, in the context of the highly prevalent HIV/AIDS epidemic in South Africa, adiposity is associated with a lack of this condition and stigmatisation [18]. In view of these positive perceptions, changing obesity enhancing behaviours likely presents a challenge. The draft South African Strategic Plan for Non-communicable Diseases, 2012–2016 suggests that consideration may be given to taxing undesirable processed foods while exempting healthier choices from taxation [20] to curb the obesity epidemic in women.

The higher prevalence of excess body weight in women than in men is a consistent observation in local national surveys [21], [22] and the SSA region [23]. The reasons for the gender difference are numerous and include socioeconomic, environmental, behavioural and cultural factors. Nutrition deprivation in childhood has been found to be a risk for obesity in women but not in men in several populations, although the physiological reasons for these variances are not well understood [24]; the lower caloric requirements in women compared to men as a result of their lower occupational and leisure time physical activity levels; lower muscle mass in women than men; and women's retention of weight gained during pregnancy, reported to be more marked among ethnic groups such as African-American women [25], are likely to be key contributors to excess body weight among women in this population.

The prevalence of overweight/obesity in men, unlike the findings in women, was comparable to that from other SSA urban centres (20.2–44.7%) [14], [15], and the prevalence of obesity was similar to the global rate of 9.8% among ≥20-year-old men in 2008 [17].

The decrease in prevalence of overweight/obesity in 25–64-year-old men between 1990 (37.3%) and 2008/09 (27.7%) was unexpected as evidence from both developed and developing regions showed that overweight/obesity usually increased over time, sometimes remained constant, but rarely decreased [14]. Globally, stable or decreasing BMI trends were found only among women in central and Eastern Europe and among men in central Africa and south Asia [17].

A possible explanation for the lower prevalence in 2008/09 in men in this study may be found in the differences in the employment rates between the samples. There were far fewer employed men in the 2008/09 (25.8%) compared to the 1990 study (73.9%), a reflection of the economic situation on the ground but could also be attributed to employed men being differentially sampled. This may have occurred because the current study was conducted during office hours while the 1990 data were collected after hours in the evenings. Overweight/obesity could possibly have been underestimated because of the significantly greater likelihood of overweight/obesity in employed compared to unemployed men present in this study.

Although overweight/obesity was not associated with employment in women, it is of interest that 53.7% of women were employed in 1990 compared to 22.3% in 2008/09 and likely demonstrates the differential influence of employment on overweight/obesity in men and women. Unemployment among women may not necessarily be associated with lower socioeconomic status as women with partners or husbands may be less likely to be the primary breadwinner.

It is unlikely that changes in cigarette smoking and alcohol use between 1990 and 2008/09 account for the different pattern of overweight/obesity in men and women. Daily smoking decreased in men but remained stable in women while alcohol use increased in both sexes.

The high burden of HIV/AIDS and tuberculosis in 2008/09 would likely contribute to weight loss in infected South Africans; this may be more pronounced in men than women because women are more likely to be screened and treated, and subsequently regain weight lost and maintain a stable weight. However, all individuals who replied in the affirmative to the question on HIV status and the use of ART during screening were excluded from the current study, as were those on tuberculosis treatment.

The positive link between wealth and employment (in men) with increased BMI and WC is in keeping with other South African studies where socioeconomic status defined by various measures including education, occupation, income and household amenities was related to increased adiposity [26], [27] as well as with studies from many SSA countries [23], [28]. In contrast, in developed regions, there is an inverse relationship between overweight/obesity and measures of socioeconomic status [15],[28]. The positive association between overweight/obesity and asset index in this study population is suggestive of the early stages of the nutrition transition. Excess weight initially presents among the affluent before progressively shifting to lower income groups, as has been demonstrated in middle-income developing and developed countries at later stages of the epidemiologic and nutrition transitions [29].

The lack of association between urbanisation and overweight/obesity in this study may be related to the definition of urbanisation as the proportion of life spent in the city, which is a blunt proxy. It may also reflect the rapidity with which new migrants to the city adopt unhealthy lifestyle behaviours that predispose to adiposity. In Tanzania, declines in physical activity, dietary changes and weight gain in rural migrants occurred within six months to a year of arrival in the city [30], [31]. Furthermore, with frequent population flows between urban and rural areas, lifestyle habits may be changing with uptake of unhealthier diets and sedentary lifestyles that contribute to obesity occurring even in rural settings.

The lack of association between physical inactivity and overweight/obesity underscores the importance of using an objective instrument to ascertain activity levels rather than the self-reported measure used in this study. Limited numeracy skills as well as assessing the time spent doing physical activity may have posed challenging during data collection [32], particularly as previous studies, including the 2003 national survey conducted using the same GPAQ questionnaire, had reported much higher levels of physical inactivity in South Africa (about 50%) [22], [32]. It is highly unlikely that activity levels would have increased so dramatically, particularly in urban environments that are not conducive to encouraging physical activity, and therefore probably reflects over-reporting.

Although more research is required on the relationship between smoking and adiposity, other cross-sectional studies, in keeping with these findings, have also found smoking to be associated with lower adiposity [33]. The marked increases in energy expenditure and the appetite suppressant properties attributed to nicotine are likely responsible for this association. However, this relationship is complex with heavy smoking associated with overweight, probably because of the greater uptake of unhealthy lifestyle behaviours in heavy compared to light smokers [33]. The mean number of cigarettes smoked in this study approximated 6.5 and 5.9, respectively, in male and female daily smokers, indicating that these were mainly light smokers which may account for the inverse relationship.

## Study Limitations

The cross-sectional study design precluded conclusions about causal associations between overweight/obesity and the associated determinants. The low sample realisation in men (64%) that is characteristic of epidemiological studies in this country necessitated higher sampling weights and a loss of precision. This was likely due to difficulties encountered in making the initial contact with potential male participants, possibly because the study was conducted during office hours. Furthermore, the latter may have resulted in selection bias; the inclusion of fewer employed compared to unemployed men may have contributed to a lower estimate of overweight/obesity in men. Considering that testing for HIV and tuberculosis infections were not undertaken, these conditions may have been undiagnosed in some participants; however, the estimates were considered to be <10%. The use of a single 24-hour dietary recall of the previous day's food intake may account for the lack of significance between total fat intake and overweight/obesity. The use of self-reported rather than objectively measured ambulation or physical activity decreased data accuracy.

## Conclusions

The extremely high prevalence of overweight/obesity among women in the black population of Cape Town in 2008/09 is concerning. The significant relationship between increasing BMI and WC, and the CVD risk factors of hypertension, diabetes and raised LDL-C highlight the adverse consequences of obesity and the need for urgent interventions in this population. Concerted prevention and control measures with a life-course approach that instils a culture of healthy lifestyle behaviours including improved diets with moderation of caloric intake and regular physical activity from childhood to older age are imperative. Moreover, in view of the positive sociocultural attitudes towards overweight/obesity, changing these ingrained perceptions remains a major challenge and needs to be addressed.
